# Supplementary material for: Validity and reliability of anthropometric equations versus Dual X-ray Absorptiometry to estimate body composition in athletes with unilateral lower-limb amputation: A pilot study
Source: PLoS One. 2025 Jul 11;20(7):e0326524. doi: 10.1371/journal.pone.0326524 (PMC12250317; doi:10.1371/journal.pone.0326524)
Supplement: Attachment — Submitted filename: Response to Reviewers.pdf [file pone.0326524.s004.pdf]

Universidad del Rosario

May 16th 2025

Editor-in-Chief

PLOS ONE

Dear Editor-in-Chief,

Thank you for the opportunity to revise our manuscript entitled " Validity and reliability of anthropometric equations versus Dual X-ray Absorptiometry to estimate body composition in athletes with unilateral lower-limb amputation: a pilot study. We appreciate the time and effort that you and the reviewers have invested in providing valuable feedback and suggestions to improve the quality of our work.

We have carefully considered all the comments raised and have revised the manuscript accordingly. Please find enclosed our point-by-point response to each of the reviewers' comments, along with a revised version of the manuscript with changes tracked as requested.

Additionally, we have initiated the data sharing process and are currently working on uploading the dataset to the public repository of Universidad del Rosario. Once finalized, the data will be openly available to support transparency and facilitate replication of our findings.

We hope that the revisions we have made satisfactorily address all concerns and improve the clarity, rigor, and overall quality of the manuscript. Please do not hesitate to contact us if further clarification is required.

Thank you again for your consideration.

Sincerely,

Laura Victoria Rivera

Laura Victoria Rivera Amézquita
Principal Professor

Universidad del Rosario, School of Medicine and Health Sciences, Rehabilitation Science Research Group, Center for the Study of Physical Activity Measurement (CEMA), Bogotá 111211, Colombia.
Carrera 24 # 63c-69. Universidad del Rosario, Bogotá, Colombia
Laurav.rivera@urosario.edu.co
+57 3163885488

Dear Editor,

The study " Validity and reliability of anthropometric equations versus Dual X-ray Absorptiometry to estimate body composition in athletes with unilateral lower limb amputation " which presents the objective " is an important study and aims at a relevant topic to be explored, however, some points need to be clarified before we proceed. Here are the key points that illustrate this clarity

Introduction

1. Problems of Structure and Cohesion

Lack of smooth transition between paragraphs: The introduction jumps from one topic to another without a clear connection. For example, after discussing the importance of body composition in Paralympic athletes, the text jumps directly to DEXA without a proper transition.

Suggestion: Add transitional phrases such as: "Given the importance of assessing body composition, accurate and accessible methods are needed. One such method is DEXA, considered the gold standard..."

Repetition of information: The introduction mentions the importance of body composition and assessment methods several times, which could be condensed to avoid redundancy.

Suggestion: Consolidate the information about the importance of body composition into a single paragraph.

Response: we have improved the overall cohesion between the paragraphs. The discussion regarding the importance of body composition assessment has been condensed for clarity and relevance, and its placement within the manuscript has been adjusted to enhance logical flow and thematic consistency.

2. Clarity and Accuracy Issues

Excessive use of quotations: The text is overloaded with quotations, which makes it difficult to read and flow. In some cases, the quotations are repeated or unnecessary.

Example: "(13– 15)(23–25)(23–25)(23–25)" is repeated and makes no sense.

Suggestion: Reduce the number of citations and group them in a more organized way. For example: "Several studies have validated the use of anthropometric equations to estimate body composition (13–15, 23–25)."

Response: the references have been properly organized and formatted in accordance with PlosOne guidelines.

Lack of explanation of technical terms: Some terms, such as "anthropometry-based predictive equations", are not explained clearly for readers who may not be familiar with the topic.

Suggestion: Add a brief explanation, such as: "Anthropometric-based predictive equations use measurements such as skinfold thickness, circumferences, and diameters to estimate body composition."

Response: the explanation of the term anthropometry-based predictive equations was added in the lines 79 to 82.

Lack of clarity in the justification for the study: The text mentions that the accuracy of anthropometric equations may be compromised in athletes with unilateral amputation, but does not explain why this is relevant or how the study intends to solve this problem.

Suggestion: Add a sentence like: "Therefore, this study seeks to analyze the concurrent validity and reliability of these equations in athletes with unilateral amputation, comparing them with the gold standard DEXA, in order to provide more accurate guidelines for this specific population."

Response: clarification about the justification of the study was included in lines 100 to 102.

3. Formatting and Style Issues

Unnecessary line numbers: Line numbers (78-101) should not appear in the final text. They appear to be leftovers from a draft or submission system.

Suggestion: Remove line numbers.

Response: Although the comment was not entirely clear, the manuscript was carefully reviewed to ensure clarity and completeness. We confirm that there are no remnants of earlier drafts in the current version.

Lack of objectivity in the objective of the study: The ultimate objective ("to analyze the concurrent validity and reliability of anthropometric equations...") is clear, but could be more specific.
Suggestion: Rephrase to something like: "This study aimed to compare the concurrent validity and reliability of anthropometric equations with DEXA in athletes with unilateral lower limb amputation, in order to evaluate their applicability in this population."

Response: The objective was reformulated as suggested in the lines 97 to 100.

4. Contextualization Problems

Lack of context about athletes with amputations: The text mentions that athletes with amputations have a higher risk of weight gain, but does not explain why this occurs or how it affects body composition.
Suggestion: Add a brief explanation, such as: "After an amputation, reduced muscle mass and decreased physical activity can lead to an increase in body fat percentage, making accurate assessment of body composition even more critical."

Response: The potential causal relationship between amputation and increased risk of weight gain is addressed in the introduction, specifically in lines 60 to 64.

Lack of discussion about the limitations of DEXA: The text mentions that DEXA is the gold standard, but does not discuss its limitations, such as cost and accessibility, which are relevant to justify the use of alternative methods.
Suggestion: Add a sentence like: "Despite its accuracy, DEXA has practical limitations, such as high cost and the need for specialized equipment, which limit its use in routine sports settings."

Response: The limitations of DEXA were further elaborated in introduction, in lines 72 to 76.

5. Grammar and Writing Problems

Inappropriate use of commas: There are several unnecessary or poorly placed commas, which impairs the flow of the text.

Example: "Specifically, in Paralympic sports, body composition measurements are a tool for monitoring the athlete's performance, health status, and functionality (7)."

Suggestion: Remove the comma after " Specifically ".

Lack of parallelism in lists: In some sentences, the items listed do not follow a parallel structure.

Example: "Estimating circumference involves using a measuring tape and taking measurements at distinct reference points, while diameters are assessed with a caliper, offering insights into the individual's bone percentage."

Suggestion: Rephrase to something like: "Estimating circumference involves using a tape measure to measure specific landmarks, while diameters are assessed with a caliper, providing information about bone structure."

Response: Unnecessary comas were removed, and the overall cohesion of the text was improved to enhance readability and clarity.

6. Problems with Citations

Repeated or disorganized quotations: The quotations "(13– 15)(23–25)(23–25)(23–25)" are repeated and disorganized.

Suggestion: Consolidate the citations into a single group, such as "(13–15, 23–25)."

Lack of recent citations: The text seems to rely on older references (e.g. 2010 or earlier). It would be interesting to include more recent studies to show that the topic is still relevant.

Suggestion: Add more recent references if available.

Response: the references have been properly organized and formatted in accordance with PlosOne guidelines. Also, we updated the manuscript by including several recent studies published between 2021 and 2025. There references contribute to a more current scientific framework and reinforce the relevance and applicability of anthropometric-

based methods in body composition assessment. Specifically, the following references have been integrated:

- Serafini S, Charrier D, Izzicupo P, Esparza-Ros F, Vaquero-Cristóbal R, Petri C, et al. Anthropometric-based predictive equations developed with multi-component models for estimating body composition in athletes. Eur J Appl Physiol. 2025 Mar;125(3):595–610: addressing recent developments in predictive euqtaions based on multicomponent models in athletes

- Rojano-Ortega D, Moya-Amaya H, Molina-López A, Berral-Aguilar AJ, Berral-de la Rosa FJ. Development and validation of a new anthropometric equation to predict skeletal muscle mass in a heterogeneous caucasian population. Sci Rep. 2024 Nov 19;14(1):28646: presenting a validated antrhopometric equation for skeletal muscle mass estimation.

- Campa F, Matias CN, Moro T, Cerullo G, Casolo A, Teixeira FJ, et al. Methods over Materials: The Need for Sport-Specific Equations to Accurately Predict Fat Mass Using Bioimpedance Analysis or Anthropometry. Nutrients. 2023 Jan 5;15(2):278: emphasizing the importance of sport-specific equations for fat mass prediction.

- Cavedon V, Sacristani F, Sandri M, Zancanaro C, Milanese C. Anthropometric prediction of DXA-measured percentage of fat mass in male karate athletes. J Sports Med Phys Fitness. 2023 Apr;63(4):558–65 and Giro R, Matias CN, Campa F, Santos DA, Cavaca ML, Duque P, et al. Development and Validation of an Anthropometric Equation to Predict Fat Mass Percentage in Professional and Semi-Professional Male Futsal Players. Nutrients. 2022 Oct 27;14(21):4514 and Dimitrijevic M, Paunovic V, Zivkovic V, Bolevich S, Jakovljevic V. Body Fat Evaluation in Male Athletes from Combat Sports by Comparing Anthropometric, Bioimpedance, and Dual-Energy X-Ray Absorptiometry Measurements. Biomed Res Int. 2022;2022:3456958: highlight the use of anthropometry and DEXA in various athletic populations.

- Totosy de Zepetnek JO, Lee JJ, Boateng T, Plastina SE, Cleary S, Huang L, et al. Test-retest reliability and validity of body composition methods in adults. Clin

Physiol Funct Imaging. 2021 Sep;41(5):417–25: providing recent evidence on the validity and reliability of body composition methods.

7. General Improvements

Add a concluding paragraph: The introduction ends abruptly with the purpose of the study. It would be helpful to add a concluding paragraph that summarizes the importance of the study and its practical implications.

Suggestion: "This study may provide valuable insights for coaches and healthcare professionals working with amputee athletes, allowing for a more accurate and accessible assessment of body composition."

Response: a concluding paragraph was added at the end of the introduction to facilitate the transition into the methods section, enhancing the logical flow of the manuscript.

METHODS

8. Sample Size:

The study included 27 athletes, which may be considered a small sample size for a validation study. This may limit the generalizability of the results.

Suggestion: Justify the sample size based on a statistical power calculation or mention that this is a pilot study that will serve as a basis for future research with larger samples.

Response: the calculation of the sample size has been added and in now described in detailed in lines 117 to 121 of the revised manuscript.

9. Lack of Description of Anthropometric Equations:

The text mentions that the equations to estimate body density (BD), fat percentage (FP), fat mass (FM), lean mass percentage (LP) and lean mass (LM) were included after an extensive literature review, but does not specify which equations were used.

Suggestion: Include a table or list of the equations used, with bibliographic references, to allow replication of the study.

Response: table 1 of the manuscript provides a detailed description of each equation used in the study, including the corresponding reference. Additionally, an explanatory text was included to specify the number of equations tested for each variable, as well as their respective references in line 183 to 185.

10. Lack of Details on Evaluator Training:

Although the text mentions that the evaluators were level 1 anthropometrists and carried out practice sessions two weeks before data collection, there are no details on how consistency between evaluators was ensured.

intraclass correlation coefficient (ICC) or other statistical method used to assess inter-rater agreement.

Response: Detailed information regarding the intra-observer technical error of measurement (TEM), along with the intraclass correlation coefficient (ICC), has been included in lines 164 to 168 of the revised manuscript.

11. Lack of Description of the Randomization Process:

It is unclear whether the order of measurements (anthropometry vs. DEXA) was randomized to avoid order bias.

Suggestion: Describe whether the measurements were taken in random order or whether there was a time interval between them to avoid interference.

Response: this clarification was incorporated in lines 126 to 130. "Anthropometric measurements were performed first, with a 40-minute interval before DEXA scan to prevent potential interference between methods. During this interval, participants were instructed to refrain from eating or drinking to endure consistent measurements conditions across both assessment techniques"

12. Lack of Discussion on Limitations of the Method:

The text does not mention the limitations inherent in the use of anthropometric equations in athletes with amputations, such as the possible asymmetric distribution of fat and muscle.

Suggestion: Include a brief discussion of how these limitations were considered in the study design.

Response: the discussion about the limitations of the use of anthropometric equations in athletes with lower-limb amputation was incorporated into the methods section (lines 173 to 177). This topic was also addressed in the discussion section in lines 715 to 739.

13. Lack of Measurement Environment Details:

The text does not describe the environment where the measurements were performed (temperature, humidity, etc.), which may affect the results, especially in bioimpedance measurements.

Suggestion: Include information about the measurement environment to ensure standardization.

Response: details about the measurement environment were included in the methods section, specifically in lines 154 to 156, to provide reproducibility of the assessment conditions.

14. Lack of Description of the Data Analysis Process:

The text does not mention how the data were analyzed to compare the concurrent validity of anthropometric equations with DEXA.

Suggestion: Include a brief description of the statistical methods used (e.g., Pearson correlation, Bland -Altman analysis, etc.).

Response: the statistical analysis section was expanded to include a clearer explanation of how concurrent validity was assessed. Specifically, the Pearson Correlation Coefficient was calculated to evaluate the association (concurrent validity) between each anthropometric equation and the DEXA results. In case where the data did not follow a normal distribution, the Spearman Correlation Coefficient was used instead (lines 230 to 233)

15. Areas Needing Improvement

Lack of Justification for the Choice of Equations:

Although the table lists several equations, there is no clear explanation as to why these specific equations were chosen for the study.
Suggestion: Include a brief justification for the selection of the equations, highlighting their applicability to athletes with amputations.

Response: the justification of the selection of the equations was included in the methods section in lines 179 to 183.

16. Lack of Information on Equation Validation:
The table does not mention whether the equations have been previously validated in similar populations (amputated athletes).
Suggestion: Include a discussion of the validity of the equations in specific populations, especially athletes with amputations.

Response: to the best to our knowledge, only one study to date evaluated the validity of anthropometric-based equations in athletes with unilateral lower-limb amputation. In response, we included table 5, which summarizes the most relevant equations along with detailed information about the sample populations in which each equation was originally validated. Furthermore, the discussion section was expanded to address the characteristics of these populations and the implications for applying these equations to our population.

17. Complexity of Equations:
Some equations are quite complex and involve multiple variables, which can make their practical application difficult.
Suggestion: Consider simplifying the equations or selecting simpler, more widely validated equations.

Response: as stated in the methods section, the selected equations were included because they are currently used by the professionals at the District Institute of Recreation and Sport (IDRD). Their inclusion is further justified by their practical applicability in field settings, as they rely on accessible and straightforward anthropometric variables such as body weight, height, skinfolds thickness, and circumferences. Additionally, these

equations are relatively simple to apply, which enhances their feasibility for use in routine assessment.

18. Lack of Consistency in Variables:

The variables used in the equations vary significantly (for example, some equations use only circumferences, while others include skinfolds and age).

Suggestion: Standardize the variables used in the equations, if possible, to facilitate comparison and application.

Response: the proposed standardization is not feasible, as our objective is to compare each equation in the form which it was originally validated. It is true that some equations rely solely on circumferences, while others incorporate variables such as skinfolds thickness, age, and race. Therefore, it is essential to use each equation in its original form to ensure methodological consistency and enhance reproducibility of our findings.

19. Lack of Information on Estimation Error:

The table does not provide information about the estimation error associated with each equation.

Suggestion: Include a column with the estimation error (e.g., standard error of the estimate) for each equation.

Response: the standard error of the estimate (of the difference between the equations and DEXA results – SED-) were calculated for each equation. This procedure was added to the methods section (lines 215 to 211). The SED values are presented in a new column in table 4, and the corresponding results have been incorporated into both, results and discussion section to support the interpretation of the equations' performance.

20. Lack of Discussion on Limitations of Equations:

The table does not discuss limitations of the equations, such as their applicability to specific populations or their sensitivity to variations in fat and muscle distribution.

Suggestion: Include a brief discussion of the limitations of the equations and how they may affect outcomes in athletes with amputations.

Response: the discussion on the applicability of the equations and their sensitivity to variations in fat and muscle distribution was expanded in the discussion section (lines 715 to 739).

21. Include a Summary Table:

Create a summary table that lists the most relevant equations and their main characteristics (variables used, estimation error, etc.).

Response: table 5 was added to the manuscript to summarize the most relevant equations evaluated in the study. It includes key characteristics such as bias compared to DEXA, Intraclass Correlation Coefficients (ICC), Correlation with DEXA, the sample in which the equation was originally validated, and the variables required for their application.

22. Add a Discussion on the Applicability of the Equations:

Include a section discussing the applicability of the equations to athletes with amputations, highlighting possible adjustments needed.

Response: the discussion on the applicability of the equations was expanded in the discussion section (lines 715 to 739).

23. Simplify the Equations:

Consider simplifying equations or selecting simpler, more widely validated equations.

Response: as stated in the methods section, the selected equations were included because they are currently used by the professionals at the District Institute of Recreation and Sport (IDRD). Their inclusion is further justified by their practical applicability in field settings, as they rely on accessible and straightforward anthropometric variables such as body weight, height, skinfolds thickness, and circumferences. Additionally, these equations are relatively simple to apply, which enhances their feasibility for use in routine assessment.

24. Include Estimation Error Information:

Add a column with the estimation error for each equation.

Response: the standard error of the estimate (of the difference between the equations and DEXA results – SED-) were calculated for each equation. This procedure was added to the methods section (lines 215 to 220). The SED values are presented in a new column in table 4, and the corresponding results have been incorporated into both, results and discussion section to support the interpretation of the equations' performance.

25. Include information on formulas for amputees regardless of the sport and their respective estimation error and population used.
Add a column with formulas used in amputees or assessment methods in this population

Response: in the discussion section, table 6 was included to present the two-and to the best of our knowledge- anthropometric equations that have been validated specifically in athletes with unilateral lower-limb amputation. This table provides relevant information, such as the equations itself, the original reference, the variables required for its application, the mean coefficient of determination (R^2), the mean absolute prediction error, and the error range reported in the original publications.

26. Areas That Can Be Improved
Wilk Test Results:
The text mentions that the Shapiro- Wilk test was used, but does not describe the results (i.e., which variables were parametric and which were nonparametric). This is important to justify the choice of subsequent tests.
Wilk test results.

Response: more specific information regarding the results of the Shapiro-Wilk, including the identification of variables with parametric and non-parametric distribution, was added to the statistical analysis section (lines 198 to 209). In this section we also detailed the statistical test used for each set of comparisons, based on the distribution characteristics determined by Shapiro-Wilk test results.

27. Justification for Using the Paired t-Test and Wilcoxon:

Although the text mentions that the paired t-test was used for parametric data and the Wilcoxon test for nonparametric data, there is no clear explanation of how this decision was made for each variable.

Suggestion: Include a brief justification for the choice of tests in each case.

Response: more specific information regarding the results of the Shapiro-Wilk, including the identification of variables with parametric and non-parametric distribution, was added to the statistical analysis section (lines 198 to 209). In this section we also detailed the statistical test used for each set of comparisons, based on the distribution characteristics determined by Shapiro-Wilk test results (paired t test or the Wilcoxon test)

28. Statistical Power Analysis:

The text does not mention whether a statistical power calculation was performed to determine the required sample size. This is important to ensure that the study has sufficient power to detect significant differences.

Suggestion: Include a discussion of the statistical power of the study.

Response: the rationale behind the calculation of the statistical power was included in the methods section (lines 117 to 121).

29. Correction for Multiple Comparisons:

Since multiple comparisons were made (e.g. between different equations and DEXA), there is an increased risk of Type I error (false positive). The text does not mention whether any correction for multiple comparisons, such as the Bonferroni correction, was made.

Suggestion: Apply a correction for multiple comparisons or justify why it was not necessary.

Response: the Bonferroni correction for multiple comparisons was applied, and the procedures, including how the corrected p-values were calculated and the thresholds considered statistically significant, is explained in the statistical analysis section (lines 209 to 213) of the methods.

Bland -Altman Test :

Bland -Altman test was used, but does not describe the results (e.g., the limits of agreement and the presence of biases).

Bland -Altman table or plot with limits of agreement and a discussion of the presence of biases.

Response: the Bland-Altman analysis and limits of agreement were calculated for all equations, and the corresponding values were included in table 4. The results of the limits of agreement, as well as the potential presence of systematic bias, are described in the results section for each equation and further discussed in the discussion section to support the interpretation of agreement with DEXA, as the reference method.

31. Use of Regression Models:

In addition to correlations and concordance tests, it could be interesting to include regression models to predict DEXA results based on anthropometric equations. This would allow a more detailed analysis of the relationship between the methods.

Suggestion: Consider including linear or non-linear regression models.

Response: A Reduced Major Axis (RMA) regression was used to analyze the relationship between the values obtained by DEXA and those predicted by each anthropometric equation. This procedure was described in the methods section under data analysis (lines 224 to 228). The intercept and the slope values from the RMA regression models were added to the table 4. The results of these models are reported in the results section and discussed in terms of proportional bias in the discussion section.

32. Interpretation of the Limits of Agreement:

Although limits of agreement are provided, there is no discussion of whether these limits are clinically acceptable. For example, a limit of agreement of -17.12% to 18.78% for body fat percentage is too broad and may not be acceptable for practical applications.

Suggestion: Include a discussion of the clinical relevance of limits of agreement, especially in athletes with amputations.

Response: the interpretation of the limits of agreement was incorporated into both, the results and discussion sections. Additionally, the clinical relevance of these limits of agreement was addressed to provide context for their practical implications in assessing the validity and applicability of the equations for athletes with unilateral lower-limb amputation.

33. Identifying Biases:

Bland -Altman plots can reveal systematic biases (e.g., whether equations consistently overestimate or underestimate DEXA values). However, there is no detailed analysis of the presence of biases.

Suggestion: Analyze and discuss the presence of systematic biases in the results.

Response: Information regarding the systematic bias of each equation was included in both, the results and discussion sections. Furthermore, the implications of this bias were critically analyzed in the discussion to support the interpretation of each equation´s accuracy and suitability for athletes with unilateral lower-limb amputation.

34. Comparison between Equations:

The graphs show the agreement of several equations, but there is no direct comparison between them to identify which are the most accurate.

Suggestion: Include a summary table that compares the mean differences and limits of agreement for all equations.

Response: Table 4 was complemented with additional information, including the slope and intercept of the RMA regression models, as well as the limit of agreement from the Bland-Altman analysis for each equation. These additions were made to facilitate a comprehensive comparison of the equations evaluated in this study and to support a more detailed interpretation of their performance relative to the DEXA reference method.

Outlier Analysis:

The graphs may reveal outliers (points outside the limits of agreement), but there is no discussion of how these outliers were handled or whether they affect the results.

Suggestion: Include an analysis of outliers and discuss their impact on the results.

Response: an analysis of the effect of the outliers on the Bland Altman analysis was included along the discussion section to better interpret their clinical impact on the agreement between each equation and DEXA measurements.

36. Correlation with Clinical Variables:

There is no analysis of how the errors in the equations correlate with clinical variables, such as the level of amputation or the type of sport practiced.

Suggestion: Include a correlation analysis between the errors in the equations and relevant clinical variables.

Response: We were not able to compare or correlate the prediction error of each equation with the type of sports or type of amputation, as the study did not have sufficient statistical power to support such subgroup analysis.
