# Supplementary material for: Validity and reliability of anthropometric equations versus Dual X-ray Absorptiometry to estimate body composition in athletes with unilateral lower-limb amputation: A pilot study
Source: PLoS One. 2025 Jul 11;20(7):e0326524. doi: 10.1371/journal.pone.0326524 (PMC12250317; doi:10.1371/journal.pone.0326524)
Supplement: Attachment — Submitted filename: ingles REVISOR v1 .docx [file pone.0326524.s002.docx]

Dear Editor,

The study " Validity and reliability of anthropometric equations versus Dual X- ray Absorptiometry to estimate body composition in athletes with unilateral lower limb amputation " which presents the objective " is an important study and aims at a relevant topic to be explored, however, some points need to be clarified before we proceed. Here are the key points that illustrate this clarity

Introduction

**1. Problems of Structure and Cohesion**

Lack of smooth transition between paragraphs: The introduction jumps from one topic to another without a clear connection. For example, after discussing the importance of body composition in Paralympic athletes , the text jumps directly to DEXA without a proper transition.

Suggestion: Add transitional phrases such as: "Given the importance of assessing body composition, accurate and accessible methods are needed. One such method is DEXA, considered the gold standard..."

Repetition of information: The introduction mentions the importance of body composition and assessment methods several times, which could be condensed to avoid redundancy.

Suggestion: Consolidate the information about the importance of body composition into a single paragraph.

**2. Clarity and Accuracy Issues**

Excessive use of quotations: The text is overloaded with quotations, which makes it difficult to read and flow. In some cases, the quotations are repeated or unnecessary.

Example: "(13– 15)( 23–25)(23–25)(23–25)" is repeated and makes no sense.

Suggestion: Reduce the number of citations and group them in a more organized way. For example: "Several studies have validated the use of anthropometric equations to estimate body composition (13–15, 23–25)."

Lack of explanation of technical terms: Some terms, such as "anthropometry-based predictive equations", are not explained clearly for readers who may not be familiar with the topic.

Suggestion: Add a brief explanation, such as: "Anthropometric-based predictive equations use measurements such as skinfold thickness, circumferences, and diameters to estimate body composition."

Lack of clarity in the justification for the study: The text mentions that the accuracy of anthropometric equations may be compromised in athletes with unilateral amputation, but does not explain why this is relevant or how the study intends to solve this problem.

Suggestion: Add a sentence like: "Therefore, this study seeks to analyze the concurrent validity and reliability of these equations in athletes with unilateral amputation, comparing them with the gold standard DEXA, in order to provide more accurate guidelines for this specific population."

**3. Formatting and Style Issues**

Unnecessary line numbers: Line numbers (78-101) should not appear in the final text. They appear to be leftovers from a draft or submission system.

Suggestion: Remove line numbers.

Lack of objectivity in the objective of the study: The ultimate objective ("to analyze the concurrent validity and reliability of anthropometric equations...") is clear, but could be more specific.

Suggestion: Rephrase to something like: "This study aimed to compare the concurrent validity and reliability of anthropometric equations with DEXA in athletes with unilateral lower limb amputation, in order to evaluate their applicability in this population."

**4. Contextualization Problems**

Lack of context about athletes with amputations: The text mentions that athletes with amputations have a higher risk of weight gain, but does not explain why this occurs or how it affects body composition.

Suggestion: Add a brief explanation, such as: "After an amputation, reduced muscle mass and decreased physical activity can lead to an increase in body fat percentage, making accurate assessment of body composition even more critical."

Lack of discussion about the limitations of DEXA: The text mentions that DEXA is the gold standard, but does not discuss its limitations, such as cost and accessibility, which are relevant to justify the use of alternative methods.

Suggestion: Add a sentence like: "Despite its accuracy, DEXA has practical limitations, such as high cost and the need for specialized equipment, which limit its use in routine sports settings."

**5. Grammar and Writing Problems**

Inappropriate use of commas: There are several unnecessary or poorly placed commas, which impairs the flow of the text.

Example : "Specifically, in Paralympic sports, body composition measurements are a tool for monitoring the athlete's performance, health status, and functionality (7)."

Suggestion: Remove the comma after " Specifically ".

Lack of parallelism in lists: In some sentences, the items listed do not follow a parallel structure.

Example : "Estimating circumference involves using a measuring tape and taking measurements at distinct reference points, while diameters are assessed with a caliper, offering insights into the individual's bone percentage."

Suggestion: Rephrase to something like: "Estimating circumference involves using a tape measure to measure specific landmarks, while diameters are assessed with a caliper, providing information about bone structure."

**6. Problems with Citations**

Repeated or disorganized quotations: The quotations "(13– 15)( 23–25)(23–25)(23–25)" are repeated and disorganized.

Suggestion: Consolidate the citations into a single group, such as "(13–15, 23–25)."

Lack of recent citations: The text seems to rely on older references (e.g. 2010 or earlier). It would be interesting to include more recent studies to show that the topic is still relevant.

Suggestion: Add more recent references if available.

**7. General Improvements**

Add a concluding paragraph: The introduction ends abruptly with the purpose of the study. It would be helpful to add a concluding paragraph that summarizes the importance of the study and its practical implications.

Suggestion: "This study may provide valuable insights for coaches and healthcare professionals working with amputee athletes, allowing for a more accurate and accessible assessment of body composition."

**METHODS**

**8. Sample Size:**

The study included 27 athletes, which may be considered a small sample size for a validation study. This may limit the generalizability of the results.

Suggestion: Justify the sample size based on a statistical power calculation or mention that this is a pilot study that will serve as a basis for future research with larger samples.

**9. Lack of Description of Anthropometric Equations:**

The text mentions that the equations to estimate body density (BD), fat percentage (FP), fat mass (FM), lean mass percentage (LP) and lean mass (LM) were included after an extensive literature review, but does not specify which equations were used.

Suggestion: Include a table or list of the equations used, with bibliographic references, to allow replication of the study.

**10. Lack of Details on Evaluator Training:**

Although the text mentions that the evaluators were level 1 anthropometrists and carried out practice sessions two weeks before data collection, there are no details on how consistency between evaluators was ensured.

intraclass correlation coefficient (ICC) or other statistical method used to assess inter-rater agreement.

**11. Lack of Description of the Randomization Process:**

It is unclear whether the order of measurements (anthropometry vs. DEXA) was randomized to avoid order bias.

Suggestion: Describe whether the measurements were taken in random order or whether there was a time interval between them to avoid interference.

**12. Lack of Discussion on Limitations of the Method:**

The text does not mention the limitations inherent in the use of anthropometric equations in athletes with amputations, such as the possible asymmetric distribution of fat and muscle.

Suggestion: Include a brief discussion of how these limitations were considered in the study design.

**13. Lack of Measurement Environment Details:**

The text does not describe the environment where the measurements were performed (temperature, humidity, etc.), which may affect the results, especially in bioimpedance measurements.

Suggestion: Include information about the measurement environment to ensure standardization.

**14. Lack of Description of the Data Analysis Process:**

The text does not mention how the data were analyzed to compare the concurrent validity of anthropometric equations with DEXA.

Suggestion: Include a brief description of the statistical methods used (e.g., Pearson correlation, Bland -Altman analysis, etc.).

**15. Areas Needing Improvement**

Lack of Justification for the Choice of Equations:

Although the table lists several equations, there is no clear explanation as to why these specific equations were chosen for the study.

Suggestion: Include a brief justification for the selection of the equations, highlighting their applicability to athletes with amputations.

**16. Lack of Information on Equation Validation:**

The table does not mention whether the equations have been previously validated in similar populations (amputated athletes).

Suggestion: Include a discussion of the validity of the equations in specific populations, especially athletes with amputations.

**17. Complexity of Equations:**

Some equations are quite complex and involve multiple variables, which can make their practical application difficult.

Suggestion: Consider simplifying the equations or selecting simpler, more widely validated equations.

**18. Lack of Consistency in Variables:**

The variables used in the equations vary significantly (for example, some equations use only circumferences, while others include skinfolds and age).

Suggestion: Standardize the variables used in the equations if possible to facilitate comparison and application.

**19. Lack of Information on Estimation Error:**

The table does not provide information about the estimation error associated with each equation.

Suggestion: Include a column with the estimation error (e.g., standard error of the estimate) for each equation.

**20. Lack of Discussion on Limitations of Equations:**

The table does not discuss limitations of the equations, such as their applicability to specific populations or their sensitivity to variations in fat and muscle distribution.

Suggestion: Include a brief discussion of the limitations of the equations and how they may affect outcomes in athletes with amputations.

**21. Include a Summary Table:**

Create a summary table that lists the most relevant equations and their main characteristics (variables used, estimation error, etc.).

**22. Add a Discussion on the Applicability of the Equations:**

Include a section discussing the applicability of the equations to athletes with amputations, highlighting possible adjustments needed.

**23. Simplify the Equations:**

Consider simplifying equations or selecting simpler, more widely validated equations.

**24. Include Estimation Error Information:**

Add a column with the estimation error for each equation.

**25. Include information on formulas for amputees regardless of the sport and their respective estimation error and population used.**

Add a column with formulas used in amputees or assessment methods in this population

**26. Areas That Can Be Improved**

Wilk Test Results :

The text mentions that the Shapiro- Wilk test was used, but does not describe the results (i.e., which variables were parametric and which were nonparametric). This is important to justify the choice of subsequent tests.

Wilk test results .

**27. Justification for Using the Paired t-Test and Wilcoxon :**

Although the text mentions that the paired t-test was used for parametric data and the Wilcoxon test for nonparametric data, there is no clear explanation of how this decision was made for each variable.

Suggestion: Include a brief justification for the choice of tests in each case.

**28. Statistical Power Analysis:**

The text does not mention whether a statistical power calculation was performed to determine the required sample size. This is important to ensure that the study has sufficient power to detect significant differences.

Suggestion: Include a discussion of the statistical power of the study.

**29. Correction for Multiple Comparisons:**

Since multiple comparisons were made (e.g. between different equations and DEXA), there is an increased risk of Type I error (false positive). The text does not mention whether any correction for multiple comparisons, such as the Bonferroni correction, was made .

Suggestion: Apply a correction for multiple comparisons or justify why it was not necessary.

**Bland -Altman Test :**

Bland -Altman test was used, but does not describe the results (e.g., the limits of agreement and the presence of biases).

Bland -Altman table or plot with limits of agreement and a discussion of the presence of biases.

**31. Use of Regression Models:**

In addition to correlations and concordance tests, it could be interesting to include regression models to predict DEXA results based on anthropometric equations. This would allow a more detailed analysis of the relationship between the methods.

Suggestion: Consider including linear or non-linear regression models.

**32. Interpretation of the Limits of Agreement:**

Although limits of agreement are provided, there is no discussion of whether these limits are clinically acceptable. For example, a limit of agreement of -17.12% to 18.78% for body fat percentage is too broad and may not be acceptable for practical applications.

Suggestion: Include a discussion of the clinical relevance of limits of agreement, especially in athletes with amputations.

**33. Identifying Biases:**

Bland -Altman plots can reveal systematic biases (e.g., whether equations consistently overestimate or underestimate DEXA values). However, there is no detailed analysis of the presence of biases.

Suggestion: Analyze and discuss the presence of systematic biases in the results.

**34. Comparison between Equations:**

The graphs show the agreement of several equations, but there is no direct comparison between them to identify which are the most accurate.

Suggestion: Include a summary table that compares the mean differences and limits of agreement for all equations.

**Outlier Analysis :**

The graphs may reveal outliers (points outside the limits of agreement), but there is no discussion of how these outliers were handled or whether they affect the results.

Suggestion: Include an analysis of outliers and discuss their impact on the results.

**36. Correlation with Clinical Variables:**

There is no analysis of how the errors in the equations correlate with clinical variables, such as the level of amputation or the type of sport practiced.

Suggestion: Include a correlation analysis between the errors in the equations and relevant clinical variables.
